# Effect of uremic toxins on hippocampal cell damage: analysis in vitro and in rat model of chronic kidney disease

**DOI:** 10.1016/j.heliyon.2021.e06221

**Published:** 2021-02-10

**Authors:** Kimio Watanabe, Emiko Sato, Eikan Mishima, Mayu Watanabe, Takaaki Abe, Nobuyuki Takahashi, Masaaki Nakayama

**Affiliations:** a Department of Nephrology, Hypertension, Diabetology, Endocrinology and Metabolism, Fukushima Medical University School of Medicine, Fukushima, 960-1295, Japan; b Department of Blood Purification, Tohoku University Graduate School of Medicine, Sendai, 980-8575, Japan; c Division of Kidney Center, St Luke's International Hospital, Tokyo, 104-8560, Japan; d Division of Clinical Pharmacology and Therapeutics, Tohoku University Graduate School of Pharmaceutical Sciences, Sendai, 980-8578, Japan; e Division of Nephrology, Endocrinology and Vascular Medicine, Tohoku University Graduate School of Medicine, Sendai, 980-8574, Japan; f Research Division of Dialysis Treatment and Chronic Kidney Disease, Tohoku University Hospital, Sendai, 980-8574, Japan

**Keywords:** Cerebro-renal interaction, Cognitive impairment, Chronic kidney disease, Uremic toxins

## Abstract

One third of the patients with chronic kidney disease (CKD) develop cognitive impairment, which is also an independent risk factor for mortality. However, the concise mechanism of cerebro-renal interaction has not been clarified. The present study examines the effects of uremic toxins on neuronal cells and analyzes the pathological condition of the brain using mouse hippocampal neuronal HT-22 cells and adenine-induced CKD model rats. Among the uremic toxins analyzed, indoxyl sulfate, indole, 3-indoleacetate, and methylglyoxal significantly decreased viability and glutathione level in HT-22 cells. The mixture of these uremic toxins also decreased viability and glutathione level at a lower dose. Adenine-induced CKD rat showed marked renal damage, increased urinary oxidative stress markers, and increased numbers of pyknotic neuronal cells in hippocampus. CKD rats with damaged hippocampus demonstrated poor learning process when tested using the Morris water maze test. Our results suggest that uremic toxins have a toxic effect on hippocampal neuronal cells and uremic CKD rats shows pyknosis in hippocampus.

## 1 Introduction

Cognitive impairment is commonly seen among patients with chronic kidney disease (CKD), with a prevalence of 16%–38% [1, 2, 3]. Cognitive dysfunction in CKD patients links to poor adherence to drug and diet therapy, and increases the time constraints faced by medical staff, number of hospitalizations, and costs [4, 5, 6, 7]. In addition, cognitive impairment has been associated with increased mortality in CKD [7]. Various factors for cognitive alteration in CKD have been discussed. Aging, diabetes, hypertension, dyslipidemia, and cardiovascular disease are known as traditional risk factors.

In CKD, a number of harmful solutes retain in blood and tissues, which are called uremic toxins. Retention of uremic toxins is involved in a variety of symptoms such as vascular calcification, renal anemia, and osteoporosis, all of which appear in CKD patients. Retention of uremic toxins is also considered as a risk factor for cognitive impairment in CKD [8, 9]. We previously reported that uremic toxins accumulate in systemic organs including brain as well as in the circulation [10]. In addition, the level of uremic toxins in cerebrospinal fluid (CSF) of Parkinson's disease is higher based on their blood level [11]. Thus, direct neuronal injury by uremic toxins could also be involved in the cognitive impairment in CKD. However, detailed mechanisms have not been clearly demonstrated. In this study, we examined the effects of uremic toxins on the neuronal cells and pathological condition of the brain using the mouse hippocampal neuronal cell line, HT-22 and adenine-induced CKD rats, which is widely used as an animal model of CKD showing obvious elevation of uremic toxins levels and kidney function impairment [12, 13, 14].

## 2 Materials and methods

### 2.1 Materials

Methylglyoxal (Sigma-Aldrich, St. Louis, MO, USA), 3-indoleacetate (IAA; FUJIFILM Wako Pure Chemical Corporation, Osaka, Japan), indole (FUJIFILM Wako Pure Chemical Corporation, Osaka, Japan), indoxyl sulfate (Sigma-Aldrich, St. Louis, MO, USA), p-cresyl sulfate (TCI Chemicals, Tokyo, Japan), hippurate (FUJIFILM Wako Pure Chemical Corporation, Osaka, Japan), creatinine (FUJIFILM Wako Pure Chemical Corporation, Osaka, Japan), methylguanidine (Sigma-Aldrich, St. Louis, MO, USA), and guanidinosuccinate (GSA; Sigma-Aldrich, St. Louis, MO, USA) were commercially obtained.

### 2.2 Cell culture

HT-22 cells were purchased from Merck KGaA (NJ, USA). Cells were maintained in normal conditions (DMEM containing 4.5 g/L glucose, 10% FBS, 100 U/mL penicillin, and 100 μg/mL streptomycin at 5% CO_2_ and 37 °C). When we performed quantification of glutathione (GSH), DMEM containing low glucose (1 g/L) and low FBS (1%) was used. In other experiments, DMEM was used at normal conditions.

### 2.3 Cell viability assay

To assess the effects of uremic toxins on hippocampal cell viability, 3-(4, 5-dimethylthiazol-2-yl)-2,5-diphenyltetrazolium bromide (MTT) assay (V13154; Thermo Fisher Scientific, MA, USA) was evaluated according to the manufacturer's protocol. Briefly, 10,000 cells seeded in 96-well plates were treated with different concentrations of uremic toxins for 24 and 48 h.

### 2.4 Quantification of GSH

GSH levels were measured using a commercial kit according to the manufacturer's protocols (GSSG/GSH Quantification kit G257, Dojindo Molecular Technologies, Inc., Japan). Briefly, 2,800,000 cells seeded in 6-well plates were treated with different concentrations of uremic toxins for 24 h.

### 2.5 Western blotting

Protein was extracted using 1 × RIPA buffer (Cell Signaling Technology, Danvers, MA, USA) containing a protease inhibitor (Roche Diagnostics K.K, Tokyo, Japan), phosphatase inhibitor cocktail (Sigma Aldrich), and 1 mM PMSF (Thermo Scientific, Waltham, MA, USA). With nuclear factor (erythroid-2-related factor)-2 (Nrf2) protein extraction, 10 μM MG132 (Sigma Aldrich) was additionally added to the above extraction buffer. Protein concentration was determined using a Quick Start protein assay (Bio-Rad Laboratories, Hercules, CA, USA), and 25 μg of protein was used in each SDS-PAGE run. 7.5% Mini-Pro TEAN Precast Gel (Bio-Rad) was used for each analysis. Protein extracts were transferred to a PVDF membrane. After blocking for 1 h, the membrane was incubated with primary antibodies (anti-Nrf2, 1:200, #14596, Cell Signaling) overnight at 4 °C. After washing, the membrane was incubated with secondary antibodies (anti-rat IgG, sc-2032, 1:5000, Santa Cruz) for 1 h at room temperature. Expression of β-actin (1:5000, sc-47778, Santa Cruz) was used as an internal control.

### 2.6 Quantitative PCR analysis

Total RNA was extracted using a RNeasy Mini kit (Qiagen, Hilden, Germany) according to the recommended protocol. Extracted RNA was reverse transcribed to cDNA using iScript Advanced cDNA Synthesis Kit for RT-qPCR (Bio-Rad Laboratories, Hercules, CA, USA) according to the recommended protocol. PCR was performed in a total volume of 2 μL containing aliquots of cDNA, 0.25 μL of 10 μM of each primer, and Luna® Universal qPCR Master Mix (New England Biolabs, MA, USA). After heating at 95 °C for 1 min, denaturation, annealing, and elongation were carried out at 95 °C for 1 min, 95 °C for 15 s, and 60 °C for 30 s, respectively. Reactions were repeated for 39 cycles. Expression of hypoxanthine phosphoribosyltransferase (*Hprt*) mRNA was used as an internal control. E. The primers were purchased from Takara (Kusatsu, Japan), and their set ID were *Il6*: MA152279, and *Hprt*: MA031262. The sequence of *p47phox* were Forward: GTCCCTGCATCCTATCTGGA and Reverse: GGGACATCTCGTCCTCTTCA.

### 2.7 Animals

Adult male Sprague-Dawley (SD) rats (224 ± 7.5g) at five weeks of age (SLC, Shizuoka, Japan) were housed under controlled environmental conditions (temperature 22 ± 1.5 °C; humidity 55% ± 5%; dark:light cycle set at 12 h:12 h, lights on at 7 a.m.). All procedures were conducted in accordance with the National Institutes of Health Guide for the Care and Use of Laboratory Animals, and the study protocols were approved by the animal ethics committee of Fukushima Medical University (approval number: 27088). The rats were divided into two groups: the control group (n = 14), and the CKD group (n = 15). The control group was fed with standard pellet food (0.8% NaCl; Nihon CLEA Japan, Inc., Tokyo, Japan), and the CKD group was fed with adenine-containing pellet food (0.5% adenine was added to standard pellet food) for three weeks (between 5 and 8 weeks of age). Blood pressure and heart rate were measured at 24 weeks of age by indirect tail-cuff method (Softron BP-2000; Tokyo, Japan). All procedures of animal experiments were conducted in accordance with the National Institutes of Health Guide for the Care and Use of Laboratory Animals, and the study protocols were approved by the animal ethics committee of Fukushima Medical University (approval number: 27088).

### 2.8 Behavioral assessment

We tested spatial learning and memory by Morris water maze experiment (MWM) according to the protocol reported previously [15]. MWM has been established as one of the most effective procedures for evaluation of spatial ability and memory related to the hippocampus, and many experiments using these methods have been reported using toxic compounds, including endogenous agents, in rodents [16, 17, 18, 19, 20]. The maze device was a circular pool with a diameter of 150 cm and depth 45 cm, which was filled with 22–24 °C water to a height of 30 cm. A platform (diameter, 10 cm), which was the evacuation area for rats, was colorless, transparent, and submerged approximately 2 cm below the water surface. Positions of the table, chair, light equipment, and experimental device were fixed in the room where the maze was located and served as spatial clues for the animals during the examination period. A video camera, positioned directly above the pool to record the swimming pattern the entire time, was attached to a computer-controlled system (Smart version 3.0, Panlab, Barcelona, Spain). Rats were initially placed in quadrants away from the platform area with their heads facing the wall. If the rats could not find an escape platform within 60 s, they were gently guided onto the platform and allowed to stay there for 20 s. For learning performance, place navigation test was conducted. Rats had five 60 s-learning trials daily in which they started in one of four quadrants randomly, with a 20 s-interval between trials. We examined their learning performance by place navigation test for four days (Phase 1 to 4). The distance travelled, escape latency to the platform, and time spent in target quadrant were calculated by averaging five trial values as indices representing learning performance. On the final day, after place navigation test, spatial memory was evaluated using probe test; the platform was removed, and rats were placed in a novel starting position. Time spent in quadrant which contained the platform was calculated as an index for spatial memory. The rats were habituated to the experimental room for 30 min before behavioral tests. After termination of each trial, they were dried with a towel and placed under a heating lamp before being returned to their home cage.

### 2.9 Collection of blood, urine, kidney, and brain samples

Twenty-four-hour urine samples were collected from rats in a metabolic cage at the age of 22 weeks. All animals were sacrificed after behavior tests were completed at the age of 24 weeks. Pentobarbital (50 mg/kg) was administered intraperitoneally for euthanasia. The abdominal cavity was then opened and as much blood as possible was collected from descending aorta. Next, it was perfused with saline. After the blood was collected, the bilateral kidneys were removed. Then, the thorax was opened and perfused transcardially with 4% paraformaldehyde in 0.1 M phosphate buffer of pH 7.4. After perfusion, the brains were collected. Five brain samples from 14 control rats and six from 15 adenine-CKD rats were stored for further histological examination.

### 2.10 Sample analysis

The following laboratory parameters were measured by a diagnostic company (SRL, Inc., Tokyo, Japan): urinary protein, urine sodium, serum creatinine, blood urea nitrogen, serum sodium, serum potassium, serum chloride, serum calcium, serum phosphate, plasma glucose, hemoglobin, hematocrit, total cholesterol, and triglyceride. Urinary 8-hydroxy-2′-deoxyguanosine (8-OHdG) and urinary isoprostane were measured by commercially available kits as previously reported [21]. Histological examination of rat brain and kidney was conducted under light microscopy with hematoxylin and eosin (H&E) staining and periodic acid-Schiff (PAS) staining, respectively. Fixed brains were cut into sections (6 μm thick) through a sagittal plane with cryostat to determine the neuronal density and neuronal nuclei using H&E staining. Kidney samples obtained from each rat were fixed in 10% buffered formalin and embedded in paraffin and serially sectioned at 2.5 μm thickness.

### 2.11 Statistical analysis

Statistical analyses were performed using IBM SPSS Statistics version 19 or JMP Pro software version 14.0.0 (SAS Institute Inc., Cary, NC, USA). Statistical comparisons of multiple groups were performed using ANOVA with the Tukey–Kramer test or Dunnett test for normally distributed variables, and the Steel-Dwass test or Steel test for non-normally distributed variables. Pearson's chi-square test was performed to evaluate the frequency of pyknosis. Values of *p* < 0.05 were considered to indicate statistical significance.

## 3 Results

### 3.1 Toxicity of uremic toxins on HT-22 cells

**Figure 1:**
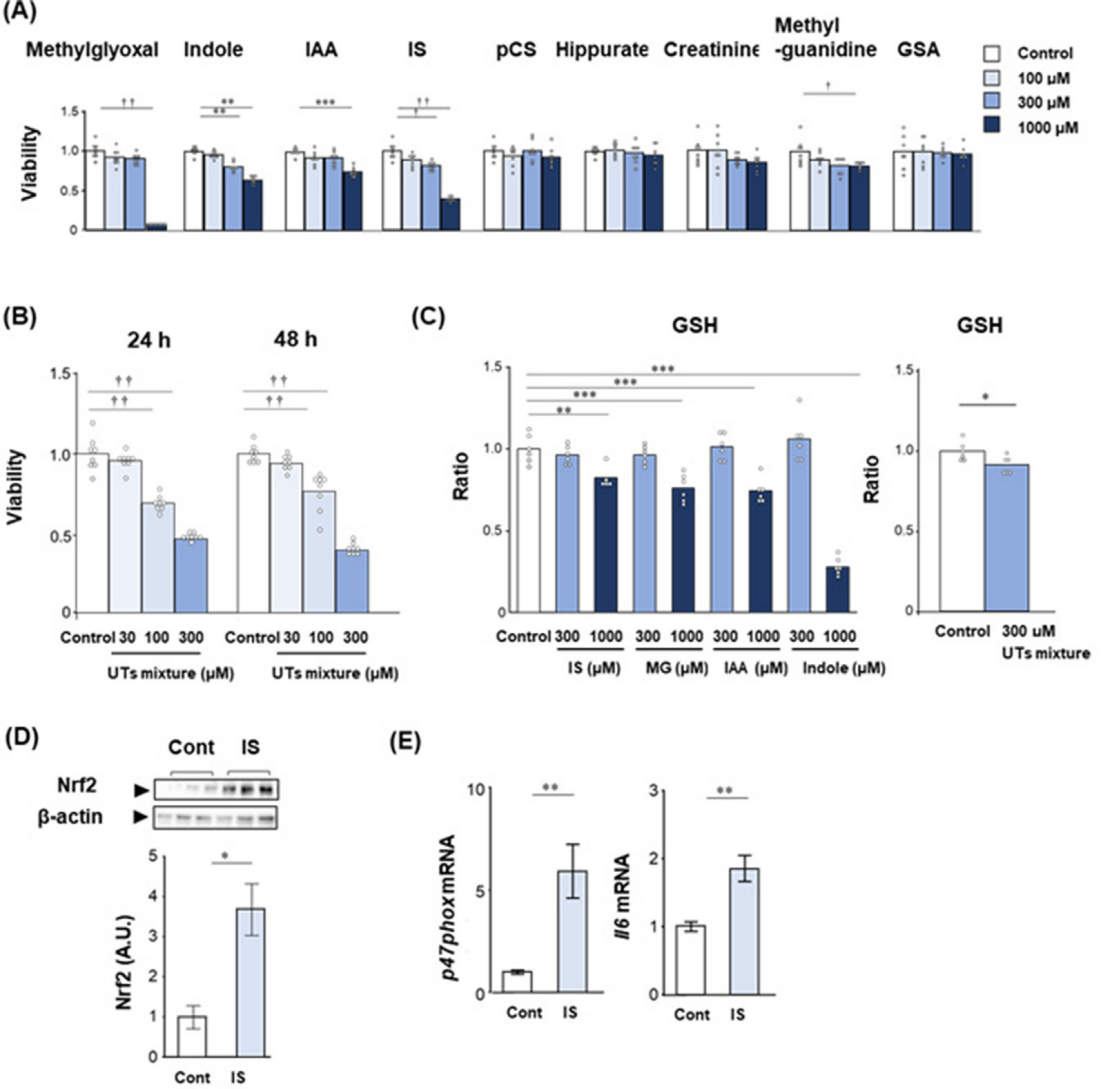
Toxicity of uremic toxins on HT-22 cells. (A) The effect of different concentrations (0, 100, 300, and 1000 μM) of uremic toxins such as methylglyoxal, indole, IAA, IS, pCS, hippurate, creatinine, methyl guanidine, and GSA on HT-22 cells for 48 h. The viability was evaluated by MTT assay. ∗*p* < 0.05, ∗∗*p* < 0.01, ∗∗∗*p* < 0.001, Dunnett test; vs. control. †*p* < 0.05, ††*p* < 0.01, Steel test; vs. control. (B) The effect of different concentrations (0, 30, 100, and 300 μM) of uremic toxins mixture containing indole, IAA, methylglyoxal, and IS. The viability was evaluated by MTT assay. ††*p* < 0.01, Steel test; vs. control. (C) The effect of different concentrations (0, 300, and 1000 μM) of uremic toxins such as indole, IAA, methylglyoxal, and IS, and uremic toxins mixture containing indole, IAA, methylglyoxal, and IS on glutathione levels. ∗∗*p* < 0.01, ∗∗∗*p* < 0.001, Dunnett test; vs. control. (D) The expression of nuclear factor (erythroid-2-related factor)-2 (Nrf2) in HT-22 by addition of indoxyl sulfate. IS: HT-22 cells incubated with 50 μM indoxyl sulfate for 1 h. Non-adjusted full western blotting images show in Figure S3 and S4. (E) The expressions of *p47phox* and *Il6* in HT-22 by addition of indoxyl sulfate. IS: HT-22 cells incubated with 50 μM indoxyl sulfate for 1 h (*Il6*) and 6 h (*p47phox*). ∗*p* < 0.05, ∗∗*p* < 0.01. Student *t*-test. Data shown is mean and data point of all samples or standard error. Abbreviations: IAA, 3-indoleacetate; IS, indoxyl sulfate; pCS, p-cresyl sulfate; GSA, guanidinosuccinate. Cont, control.

We examined the toxicity of uremic toxins including methylglyoxal, indole, IAA, indoxyl sulfate, p-cresyl sulfate, hippurate, creatinine, methylguanidine, and GSA, which have been reported for their involvement in cognitive dysfunction [9], on HT-22 cells using MTT assay. In the present study, we have used the 100 μM, 300 μM, and 1000 μM of uremic toxins were used, because the maximal concentration of indoxyl sulfate in renal failure is about 1.1 mM [22]. Indoxyl sulfate, methylglyoxal, indole, and IAA significantly deceased the viability of HT-22 cells at 300 μM or 1000 μM concentrations in both 24 h and 48 h (Figure 1A). Methylguanidine is toxic to HT-22 cells when incubated for 48 h. In addition, we examined the toxic effects of uremic toxins mixture containing the same concentrations of methylglyoxal, indole, IAA, and indoxyl sulfate. The uremic toxins mixture significantly decreased the viability of HT-22 cells at 100 μM and 300 μM concentrations in both 24 h and 48 h (Figure 1B). Furthermore, we measured the effect of uremic toxins and its mixture on cellular glutathione levels. Indoxyl sulfate, methylglyoxal, IAA, and indole significantly decreased the glutathione levels. The uremic toxins mixture significantly decreased glutathione level in HT-22 at a lower dose of 300 μM (Figure 1C). We next examined the expression of Nrf2, which is a main transcription activator in response to oxidative stress [23], stimulated by 50 μM indoxyl sulfate in HT-22 cells. The expression of Nrf2 was significantly increased by indoxyl sulfate stimulation compared to control in HT-22 cells (Figure 1D). In addition, the mRNA expression of *p47phox* and *Il6*, which are a regulatory subunit of the NADPH oxidase isoform 2 and an inflammation marker, respectively, were significantly increased by stimulated with 50 μM indoxyl sulfate in HT-22 cells (Figure 1E).

### 3.2 Pathological condition of the kidney and the brain in adenine-induced CKD rat

**Figure 2:**
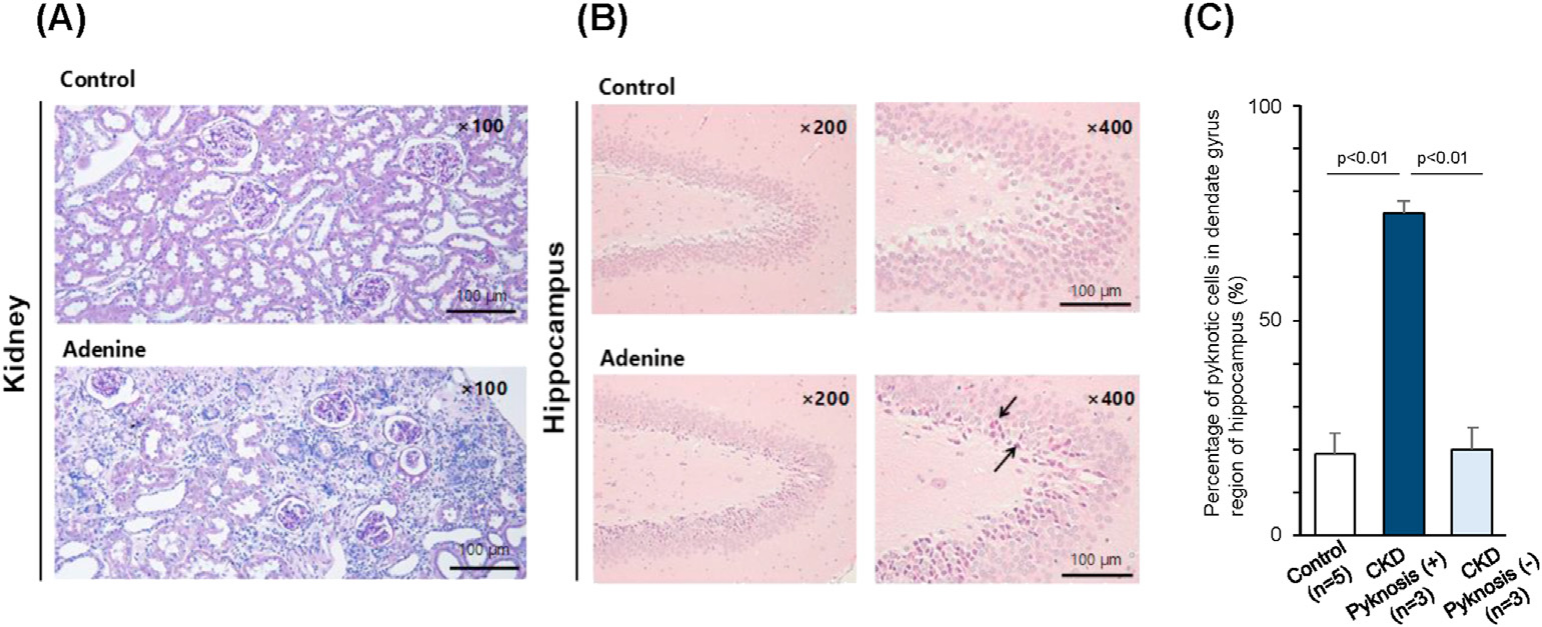
Histological findings of the kidney and the brain. (A) Microscopic examination of the kidney in periodic acid-Schiff (PAS) stain. Representative image of the kidney from the control rats (upper panel) and the adenine CKD rats (lower panel). Scale bars: 100 μm. (B) Hematoxylin and eosin (H&E)-staining of the hippocampus region. Representative image of the cornu ammonis (CA) of the hippocampus from the control rats (upper panel) and the adenine CKD rats (lower panel). Arrows: pyknotic neuronal cell bodies. Scale bars: 100 μm. (C) Percentage of pyknotic cells in dendate gyrus region if hippocampus, One-way analysis of variance (ANOVA) with repeated measures was performed with a post-hoc analysis using a simple effects analysis with a Bonferroni adjustment. F = 40.315, p < 0.01.

**Figure 3:**
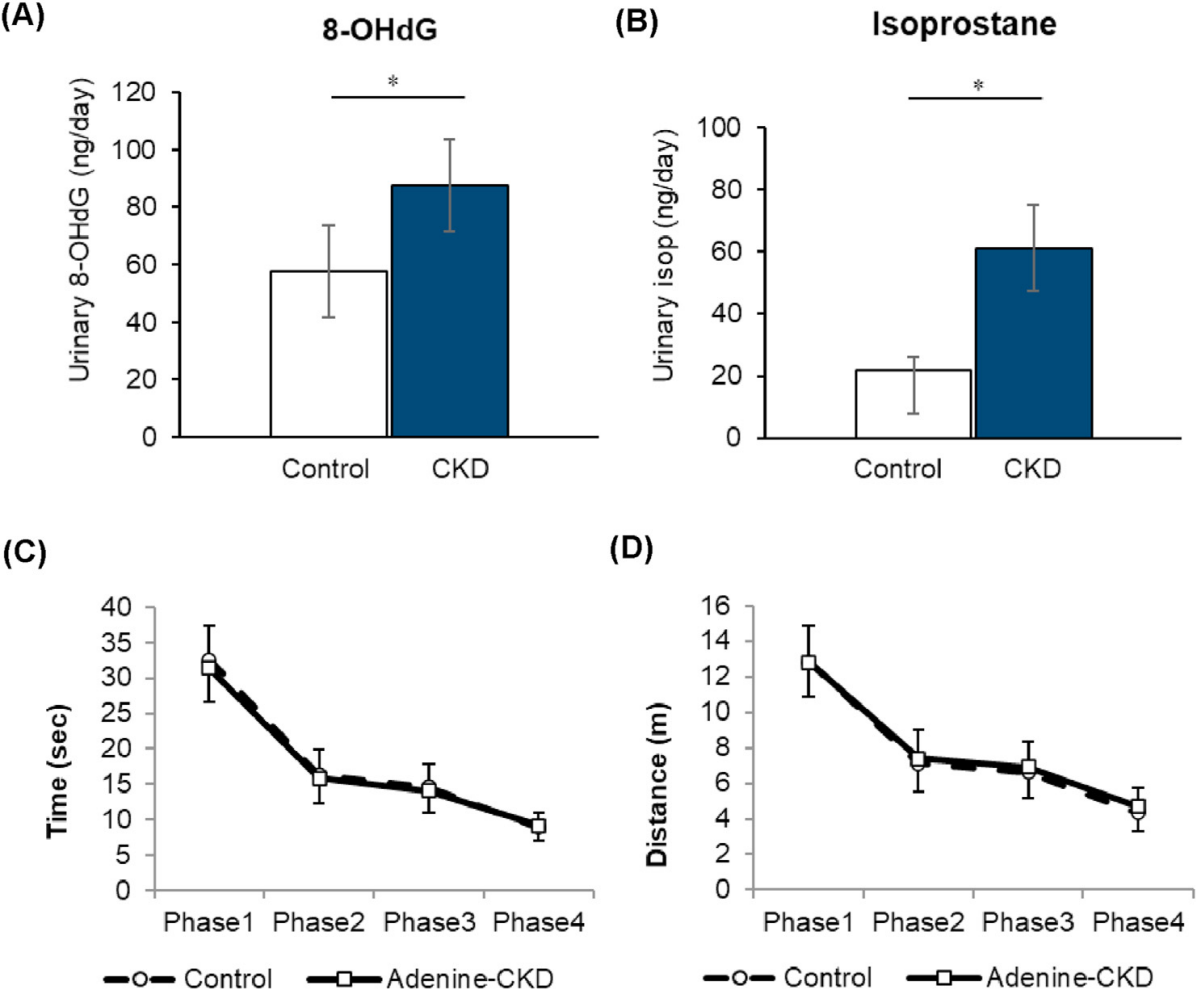
Results of urinary oxidative stress marker and spatial learning and memory testing by Morris water maze experiment (MWM). (A) Urinary 8-hydroxy-2′-deoxyguanosine (8-OHdG) and (B) urinary isoprostane. (C) Latency to find platform in place navigation test. (D) Distance to find platform in place navigation test. Data are presented as mean ± SEM. ∗p < 0.05 among the groups.

To examine the difference on brain in renal failure, we used adenine-induced CKD rat, in which uremic toxins were retained in the body [10]. Characteristics of control rats and CKD rats are shown in Table 1. Statistically significant differences were seen in urine volume, urinary protein, blood urea nitrogen, serum creatinine, hemoglobin, hematocrit, and total cholesterol among the groups. These findings are consistent in the CKD model. Histologically, CKD rats showed prominent renal damage with tubulointerstitial fibrosis (Figure 2A). During analysis of the brain sections, pyknotic neuronal cells were found in a region of hippocampus in some of the CKD rats (3 in 6), which was not seen in control rats (0 in 6) (Figure 2B). The level of urinary 8-OHdG and isoprostane, which are oxidative stress markers, was higher in CKD rats (Figure 3A and 3B). In behavior testing by Morris water maze experiment (MWM), the groups did not differ in cognitive functioning during the study (Figure 3C and 3D). Place navigation test also revealed no differences in learning process among the groups. In addition, there was no significant correlation between urinary markers and cognitive function tested data by MWM (Supplemental Figure S1 and Figure S2).

**Table 1:** Vital sings and general blood and urine test.

Parameters	Control (n = 14)	Adenine (n = 15)	P value
BW (g)	584.0 ± 57.9	554.0 ± 31.8	0.234
SBP (mmHg)	112.2 ± 20.3	112.6 ± 15.1	0.849
DBP (mmHg)	84.4 ± 15.5	77.2 ± 17.5	0.338
HR (bpm)	360.3 ± 37.8	367.3 ± 35.4	0.838
Urine volume (mL/day)	11.2 ± 2.5	35.9 ± 8.5	<0.001
Urine protein (mg/day)	16.1 ± 10.9	53.3 ± 25.7	<0.001
Urine Na (mEq/day)	0.54 ± 0.23	0.51 ± 0.19	0.747
Brain volume (g)	2.16 ± 0.04	2.20 ± 0.10	0.181
BUN (mg/dL)	20.9 ± 2.7	55.4 ± 15.5	<0.001
SCr (mg/dL)	0.35 ± 0.03	0.68 ± 0.17	<0.001
UA (mg/dL)	1.48 ± 1.34	2.90 ± 2.97	0.132
Serum Na (mEq/L)	143.2 ± 1.8	144.3 ± 1.7	0.077
Serum K (mEq/L)	4.3 ± 0.5	5.3 ± 1.9	0.057
Serum Cl (mEq/L)	104.7 ± 2.3	108.0 ± 4.3	0.029
Serum Ca (mg/dL)	10.6 ± 0.2	10.8 ± 0.8	0.78
Serum Pi (mg/dL)	6.1 ± 0.9	6.6 ± 1.5	1
PG (mg/dL)	194.9 ± 48.2	222.2 ± 95.7	0.343
Hb (g/dL)	14.0 ± 0.5	12.5 ± 1.3	<0.001
Hct (%)	43.9 ± 1.6	39.4 ± 2.9	<0.001
T.Chol (mg/dL)	71.3 ± 10.9	105.0 ± 21.9	<0.001
TG (mg/dL)	76.0 ± 36.8	82.9 ± 36.2	0.505

Data expressed as the mean ± standard deviation of the mean. BW, body weight; SBP, systolic blood pressure; DBP, diastolic blood pressure; HR, heart rate; Na, sodium; BUN, blood urea nitrogen; SCr, serum creatinine; UA, uric acid; K, potassium; Cl, chloride; Ca, calcium; Pi, phosphate; PG, plasma glucose; Hb, hemoglobin; Hct, hematocrit; T.Chol, total cholesterol; TG, triglyceride.

**Figure 4:**
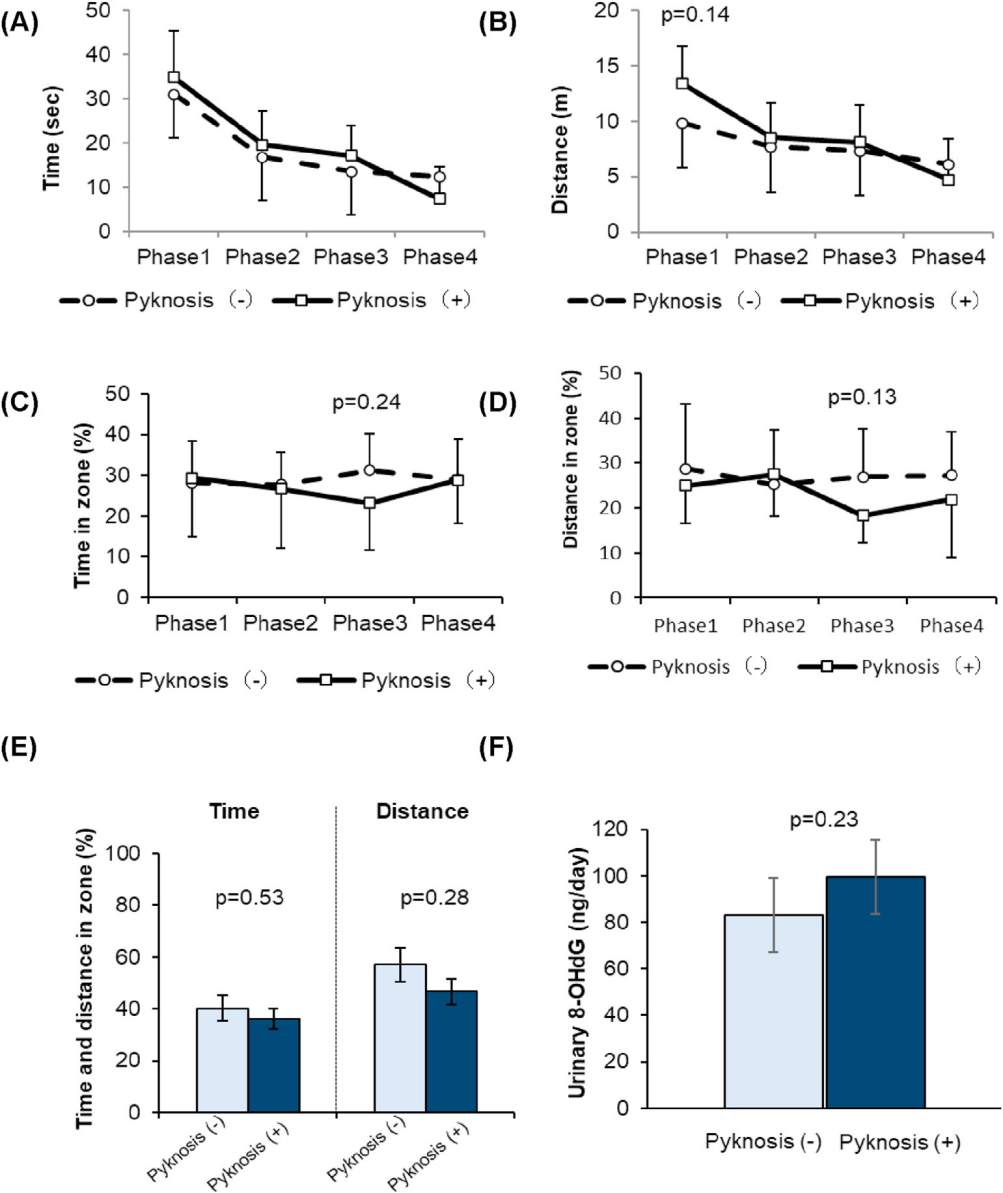
Results of urinary oxidative stress marker and Morris water maze experiment (MWM) in adenine-fed rats in which brain tissue was examined histologically. (A) Latency to find platform in place navigation test. (B) Distance to find platform in place navigation test. (C) Time spent in platform zone during the learning process. (D) Travel distance in platform zone during the learning process. (E) Time and distance spent in platform zone in probe test. (F) Urinary 8-hydroxy-2′-deoxyguanosine (8-OHdG). Data are presented as mean ± SEM.

In addition, we retrospectively analyzed by dividing the CKD rats into CKD with and without pyknosis group by percentage of pyknotic cells. The area of pyknotic cells extended throughout the hippocampus in CKD with pyknosis group. We defined more than 50% pyknotic cells were observed in the hippocampus as CKD with pyknosis group. Cells with a distinct nucleus and nucleolus were regarded as intact neurons. Percentage of pyknotic cells in dentate gyrus were 19 ± 5% in Control group (n = 5), 75 ± 3% in CKD with pyknosis group (n = 3), and 20 ± 5% in CKD without pyknosis group (n = 3) (Figure 2C). No significant change was found in other areas of hippocampus, such as CA1 and CA3, also in cerebral cortex, amygdala and thalamus. We analyzed the results of MWM in i) CKD with pyknosis group, and ii) CKD without pyknosis. Characteristics of CKD rats with pyknosis and without pyknosis are shown in Table 2. In CKD rats with pyknosis, systolic blood pressure and pulse pressure tended to be higher compared to those of CKD rats without pyknosis. Place navigation test in phase 1 to 3 did not differ between CKD with pyknosis and without pyknosis (Figure 4A), and there was a slightly longer, though not significant, distance taken to find the platform in learning process in CKD rats with pyknosis (Figure 4B, *p* = 0.14), suggesting a little bit poor learning process. In phase 3 and 4 of MWM, time spent did not differ between CKD with pyknosis and without pyknosis (*p* = 0.24) and travel distance (*p* = 0.13) in the platform zone slightly poorer, but not significant, in CKD rats with pyknosis (Figure 4C and 4D). In the probe test, time spent and moving distance in platform zone were not different between CKD rats with pyknosis and CKD rats without pyknosis (Figure 4E). The level of urinary 8-OHdG was a slightly higher, but not significant, in CKD rats with pyknosis compared to that of CKD rats without pyknosis (*p* = 0.23, Figure 4F).

**Table 2:** Characteristics of adenine-induced CKD rats with or without a finding of pyknosis cells in the brain hippocampus region.

Parameters	Pyknosis (-) (n = 3)	Pyknosis (+) (n = 3)
BW (g)	525 ± 35.3	558 ± 24.7
SBP (mmHg)	98.0 ± 24.0	109.3 ± 3.2
DBP (mmHg)	68.3 ± 24.4	60.3 ± 6.7
Pulse pressure (mmHg)	29.8 ± 0.4	49.0 ± 9.9
HR (bpm)	409 ± 33.2	369 ± 15.6
Urine protein (mg/day)	37.2 ± 0.3	50.3 ± 13.8
BUN (mg/dL)	62.2 ± 13.3	62.1 ± 13.6
SCr (mg/dL)	0.76 ± 0.26	0.64 ± 0.08
Serum Na (mEq/L)	145 ± 1.4	144 ± 1.4
Serum K (mEq/L)	4.5 ± 0.1	5.0 ± 0.3
Serum Cl (mEq/L)	109 ± 5.7	111 ± 9.2
Serum Ca (mg/dL)	10.4 ± 0.1	10.4 ± 0.1
Serum Pi (mg/dL)	6.7 ± 1.2	5.5 ± 0.7
Hb (g/dL)	12.3 ± 1.7	12.7 ± 0.1
Hct (%)	38.6 ± 2.2	40.5 ± 2.7

Data expressed as the mean ± standard deviation of the mean. BW, body weight; SBP, systolic blood pressure; DBP, diastolic blood pressure; HR, heart rate; BUN, blood urea nitrogen; SCr, serum creatinine; Na, sodium; K, potassium; Cl, chloride; Ca, calcium; Pi, phosphate; Hb, hemoglobin; Hct, hematocrit.

## 4 Discussion

In this study, we found that uremic toxins showed direct toxic effects on hippocampal neuronal cell line, and increased numbers of pyknotic neuronal cells were observed in the hippocampus of adenine-induced CKD model rats with increase in urinary oxidative stress markers. Our results suggest that uremic toxins have a toxic effect on hippocampal neuronal cells and a part of adenine-induced rats have a pyknotic neuronal cells.

In CKD, circulating uremic toxins accumulate due to renal dysfunction. In addition, gut microbiome is changed by influx urea and other retained toxins in CKD condition. The altered gut microbiome resulted in the increase of bacteria that produce uremic toxins such as indoxyl sulfate, and p-cresyl sulfate [24]. The maximal concentration of indoxyl sulfate in renal failure is about 1.1 mM, which is 366 times higher than normal concentration [22]. The maximal concentration of methylglyoxal and IAA in renal failure is 3 times and 518 times higher than normal condition, respectively [22]. Our previous studies have shown that uremic toxins that accumulate in blood transfer and accumulate in systemic organs including the brain [10]. Indoxyl sulfate and guanidino compounds undergo efflux transport in the blood-brain barrier/blood-cerebrospinal fluid barrier via organic anion transporter 3 (OAT3) and organic cation transporter 3 (OCT3), respectively [25, 26, 27, 28]. The recent paper from Bobot et al. indicated that uremic toxic blood brain barrier disruption mediated by aryl hydrocarbon receptor activation leads to cognitive impairment in CKD model rats [29]. The accumulation of uremic toxins is thought to be the cause of cognitive impairment [9, 30]. Several animal studies have reported the toxicity of uremic toxins on brain. The recent paper from Karbowska et al. reported that chronic administration of indoxyl sulfate lead to the accumulation of indoxyl sulfate to brainstem, cerebellum, and striatum with hippocampus, and contributes to the impairment of spatial memory and motor coordination in rat [31]. Guanidino compounds (creatinine, guanidine, GSA, and methylguanidine) have been reported to have neurotoxic effects [32]. Hippocampal injection of GSA in mice affected cognitive performance, activity, and hippocampal volume [33]. Administration of IAA to pregnant mice induces apoptosis in embryonic neuroepithelium, decreases formation of neurons, and leads to microencephaly in the fetuses [34]. Exposure of rat hippocampal neurons to methylglyoxal decreased glutathione levels [21]. In clinical studies, circulating indoxyl sulfate level is associated with lower cognitive function in CKD patients [35, 36], and serum IAA is associated with cognitive impairment in hemodialysis patients [37]. Recently, Lin et al. reported that indoxyl sulfate can induce neurotoxicity in patients with CKD and the pathogenesis involves cell apoptosis via oxidative stress induction in human astrocytes [38]. These findings coincide with those of the present study, proving that the accumulation of uremic toxins may trigger the pathogenesis of cognitive dysfunction in CKD, and uremic toxins in the brain might be a therapeutic target for ameliorating cognitive impairment in CKD.

In this study, we did not find the significant difference in cognitive performance tests by MWM. Previous animal studies evaluate the cognitive dysfunction in CKD rats or mice by MWM [18, 39], however, it has been reported that swimming speed affects the results of the water maze test [40]. Thus, one possible reason for this that, adenine-induced CKD rats may swim faster than control rats due to reduced body weight in the present study. To resolve this issue, we need to test a model of renal failure in which body weight is unchanged. We confirmed that CKD rats with pyknosis have slightly poor learning process, but not significant, increased oxidative stress makers, high systolic blood pressure, and high pulse pressure. We think that this result could be partially explained by the retention of uremic toxins. Oxidative stress caused by uremic toxins is recognized as one of the non-traditional risk factors for neurological complications in CKD patients [41, 42, 43]. Our data indicated that uremic toxins decrease GSH level in HT-22 cells and upregulate oxidative stress in CKD model rats. Therefore, oxidative stress in hippocampus induced by uremic toxins might contribute to cerebro-renal interaction. However, there are several limitations in the present study. The number of subjects analyzed pyknosis was small and we could not demonstrate a significant deterioration of cognitive function in CKD model rat. Thus, we need to examine whether treatment to reduce uremic toxins in the body could improve pyknosis and cognitive function.

## 5 Conclusion

In conclusion, oxidative stress in hippocampus induced by uremic toxins is one of the factors of cognitive dysfunction. Thus, therapeutic targets for uremic toxins in the brain may protect CKD patients from brain damage. Pathogenic significance of these mechanisms for cerebro-renal interaction requires further investigation.

## Declarations

### Author contribution statement

Kimio Watanabe, Emiko Sato: Conceived and designed the experiments; Performed the experiments; Analyzed and interpreted the data; Wrote the paper.

Eikan Mishima, Masaaki Nakayama: Conceived and designed the experiments; Analyzed and interpreted the data; Wrote the paper.

Mayu Watanabe: Performed the experiments; Wrote the paper.

Takaaki Abe, Nobuyuki Takahashi: Analyzed and interpreted the data; Wrote the paper.

### Funding statement

This work was supported by 10.13039/501100001691JSPS KAKENHI [Grant Numbers 15K19461, 19K08669], JSPS KAKENHI [Grant Number 23591196] and JADP [Grant Number 2013-1].

### Data availability statement

Data will be made available on request.

### Declaration of interests statement

The authors declare no conflict of interest.

### Additional information

No additional information is available for this paper.

## Appendix A Supplementary data

Supplemental_Heliyon-format Supplemental_Heliyon-format
