# Supplementary material for: Effect of uremic toxins on hippocampal cell damage: analysis in vitro and in rat model of chronic kidney disease
Source: Heliyon. 2021 Feb 10;7(2):e06221. doi: 10.1016/j.heliyon.2021.e06221 (PMC7892929; doi:10.1016/j.heliyon.2021.e06221)
Supplement: Supplemental_Heliyon-format [file mmc1.docx]

**Evaluation of the effect of uremic toxins on hippocampal cell damage in vitro and cognitive function in chronic kidney disease rat**

**Kimio Watanabe^1,2,3^*, Emiko Sato^4,5^*, Eikan Mishima^5^, Mayu Watanabe^4^, Takaaki Abe^5^, Nobuyuki Takahashi^4,5^ and Masaaki Nakayama^1, 3, 6^**

^1.^ Department of Nephrology, Hypertension, Diabetology, Endocrinology and Metabolism, Fukushima Medical University School of Medicine, Fukushima, 960-1295, Japan

^2.^ Department of Blood Purification, Tohoku University Graduate School of Medicine, Sendai, 980-8575, Japan

^3.^ Division of Kidney Center, St Luke's International Hospital, Tokyo, 104-8560, Japan

^4.^ Division of Clinical Pharmacology and Therapeutics, Tohoku University Graduate School of Pharmaceutical Sciences, Sendai 980-8578, Japan

^5.^ Division of Nephrology, Endocrinology and Vascular Medicine, Tohoku University Graduate School of Medicine, Sendai 980-8574, Japan

^6.^ Research division of Dialysis treatment and Chronic Kidney Disease, Tohoku university hospital, Sendai, 980-8574, Japan

A)

A)

B)

B)

C)

Figure S1. The correlation between urinary markers 8-hydroxy-2'-deoxyguanosine (8-OHdG) and cognitive function tested data by Morris water maze experiment (MWM). A) Correlation graph between urinary 8-OHdG and cognitive function tested data in both adenine-induced CKD rat (●) and control rat (〇). B) Correlation graph between urinary 8-OHdG and cognitive function tested data in control rat (〇). C) Correlation graph between urinary 8-OHdG and cognitive function tested data in adenine-induced CKD rat (●).

A)

A)

C)

B)

Figure S2. The correlation between urinary markers isoprostane and cognitive function tested data by Morris water maze experiment (MWM). A) Correlation graph between urinary isoprostane and cognitive function tested data in both adenine-induced CKD rat (●) and control rat (〇). B) Correlation graph between urinary isoprostane and cognitive function tested data in control rat (〇). C) Correlation graph between urinary isoprostane and cognitive function tested data in adenine-induced CKD rat (●).


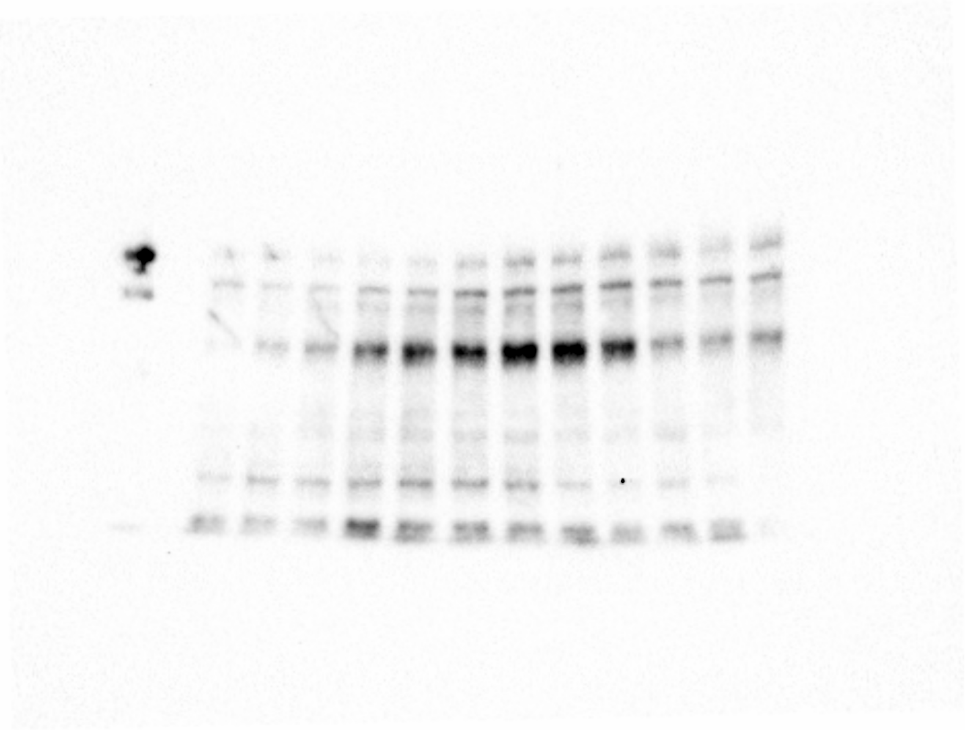


Figure S3. Non-adjusted full western blotting images of Nrf-2. Highlighted lanes of unedited gel correspond to Figure 1D.


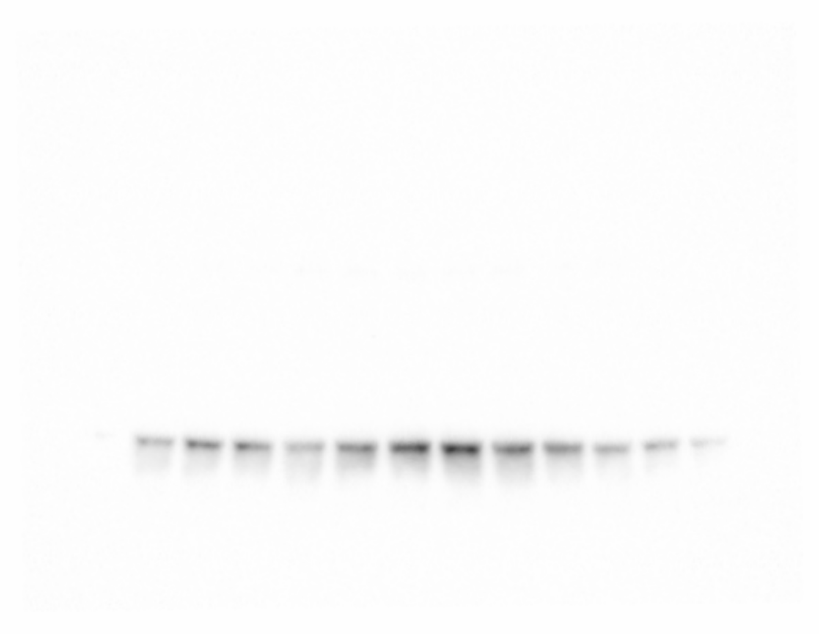


Figure S4. Non-adjusted full western blotting images of β-Actin. Highlighted lanes of unedited gel correspond to Figure 1D.
